# Information Theory Solution Approach to the Air Pollution Sensor Location–Allocation Problem

**DOI:** 10.3390/s22103808

**Published:** 2022-05-17

**Authors:** Ziv Mano, Shai Kendler, Barak Fishbain

**Affiliations:** 1 Faculty of Civil & Environmental Engineering, Technion—Israeli Institute of Technology, Haifa 3200003, Israel; ziv.mano@campus.technion.ac.il (Z.M.); skendler@technion.ac.il (S.K.); 2 Environmental Physics Department, Israel Institute for Biological Research, 24 Lerer St., Ness Ziona 7410001, Israel

**Keywords:** air pollution, environmental monitoring networks, location–allocation models, sensors’ array, information theory

## Abstract

Air pollution is one of the prime adverse environmental outcomes of urbanization and industrialization. The first step toward air pollution mitigation is monitoring and identifying its source(s). The deployment of a sensor array always involves a tradeoff between cost and performance. The performance of the network heavily depends on optimal deployment of the sensors. The latter is known as the location–allocation problem. Here, a new approach drawing on information theory is presented, in which air pollution levels at different locations are computed using a Lagrangian atmospheric dispersion model under various meteorological conditions. The sensors are then placed in those locations identified as the most informative. Specifically, entropy is used to quantify the locations’ informativity. This entropy method is compared to two commonly used heuristics for solving the location–allocation problem. In the first, sensors are randomly deployed; in the second, the sensors are placed according to maximal cumulative pollution levels (i.e., hot spots). Two simulated scenarios were evaluated: one containing point sources and buildings and the other containing line sources (i.e., roads). The entropy method resulted in superior sensor deployment in terms of source apportionment and dense pollution field reconstruction from the sparse sensors’ network measurements.

## 1. Introduction

In recent years, the negative impact of air pollution on health and climate change has become a major environmental issue. According to the World Health Organization, air pollution has emerged as the deadliest form of pollution and the fourth leading risk factor for premature deaths worldwide, accounting for about seven million deaths in 2012 [1] with a toll of about US $225 billion in lost labor income in 2013 [2,3]. Hence, controlling and monitoring air pollution is crucial. Routine monitoring is typically done by standardized air quality monitoring (AQM) stations spread thinly due to their size and cost [4]. Therefore, the effective deployment of AQM is crucial.

Advances in sensory and communication technologies have made the deployment of portable and relatively low-cost Micro-Sensing air pollution Units (MSUs) feasible. These sensors can be spread more densely and provide higher spatial resolution data. Recent studies evaluating these sensors in laboratory and field trials have shown that these units are less accurate than standard laboratory equipment or AQM stations; however, their sheer number makes it possible to effectively capture air pollution spatiotemporal variability [5,6,7,8]. While these sensors are becoming increasingly available compared to AQM, procuring, maintaining, and operating many MSUs is still a demanding task. Hence, sensor networks remain limited in size, so even for MSU networks, an optimal deployment strategy is critical.

Placing sensors in optimal locations so that the sensory network provides valuable environmental information is known as the *location–allocation* problem and has attracted considerable attention for many years [9,10,11,12]. The sensor location–allocation problem has also been studied for water resource management [13,14,15], structural health monitoring [16], soil contamination [17], and many other domains. While different systems pose different challenges, the sensor location–allocation problem can be viewed as a case of choosing the best subset of sensor locations from a set of candidates that results in a desirable outcome under budget constraints, which usually dictate the number of sensors and their properties. Formulating the location–allocation problem in this fashion serves to cast it as the well-known knapsack problem [11]. Thus, the location–allocation problem is NP-hard. Since there is no computationally efficient solution, heuristic approaches are often applied. 

There are many examples of such heuristics. Zou et al. [18] utilized sensor deployment spatial proximity models and the theoretical reliability of Gaussian dispersion processes of air pollutants to build a Gaussian weighting function-aided proximity model (GWFPM). Li et al. [19] used inverse distance weight (IDW) interpolation coupled with a geographic information system (GIS) to assess the particle matter (PM) dense pollution field for the placement of sensors in locations with the highest pollution (i.e., hot spots). Another method that capitalizes on GIS capabilities was presented by Alsahli and Harbi [20], where land use was inferred from GIS data, and sensors were deployed based on a greedy algorithm that traded off highly polluted with highly populated areas. However, the use of GIS systems requires detailed information on the target region. Often, these data are unavailable or grossly inaccurate [21]. Furthermore, placing the sensors in locations where the substance recorded by the sensors is the highest or near populated areas does not guarantee optimality in terms of pollution field reconstruction and source apportionment.

Optimization-based methods have also been used to solve the location–allocation problem. Two main problems have been addressed: optimizing network operations through connectivity and coverage [22] and optimizing air-quality sensing. For the latter, Boubrima et al. cast the optimization problem as a minimum cost problem that finds optimal sensors and sink locations, ensuring air pollution coverage and network connectivity [23,24,25]. Zoroufchi-Benis et al. [26] defined the optimization problem as a minimum fitness problem with multi-objective functions with the aim of ensuring maximum coverage, continuity of the coverage area, the least overlap among coverage areas, maximum detection of violations over ambient air standards, and sensitivity of monitoring stations to emission sources. Al-Adwani et al. [27] formulated the monitoring cost minimization problem as a minimum set cover [28], where the maximum number of overlapping points in space was correlated with the maximum number of peaks. In this work, the pollution dispersion model consisted of a Gaussian plume model to describe the dispersion of continuous emissions in steady-state conditions and a Gaussian puff model that simulated instantaneous emissions. The findings showed that the cost of monitoring could be reduced without a concomitant loss of information by minimizing the number of stations. Kumar et al. [29] presented a deterministic spatial sampling design to capture intra-city variability in air pollution. Their objective was to draw a sample of households that best represented the spatial distribution of ambient air pollution while maximizing the variance in the preliminary estimates of air pollution with the minimum number of sample sites. The algorithm ensured that the sample sites were informative for addressing inferences by emphasizing certain population or environmental characteristics. Kanaroglou et al. [10] developed a methodology for selecting monitoring sites based on spatial variations in air pollution and the distribution of addresses over the target area. The network density increased with concentration variability and population. The method specified a continuous demand surface (for monitoring) over the area. Lerner et al. [11] cast the air pollution location–allocation problem as the knapsack problem, where a given sensor’s utility in a given location was inferred from the sensor’s physicochemical characteristics and land-use analysis. 

These works all dealt with the three main factors that affect the solution of the location–allocation problem: land use, meteorological conditions, and pollution signal characteristics. The latter has mostly been considered in terms of the signal’s extreme points, i.e., hotspots. However, no previous work has attempted to associate these three factors in one framework to provide a more comprehensive solution. 

This paper presents a new approach to the location–allocation problem, which takes the topography of the observed area and its meteorology, as well as its expected pollution signal characteristics, into account. This is done by utilizing Lagrangian atmospheric dispersion models and information theory to solve the location–allocation problem. Sensors are regarded as information sources that vary due to the nature of pollution dispersion and variations in climatic conditions. The sensors are placed in a configuration that maximizes the amount of information, i.e., the joint information according to Shannon entropy, and minimizes redundancy, i.e., the mutual information.

## 2. Materials and Methods

### 2.1. Simulation Study

A simulation was carried out to evaluate sensor deployment strategies. A GRAZ Lagrangian (GRAL) dispersion model, combined with a Prognostic Wind Field Model GRAMM [30,31] was used to facilitate the examination of a wide range of scenarios differing in source characteristics and environmental conditions. 

GRAL is an open-source air pollution simulator developed by the Graz University of Technology (TUG) and the Government of Styria, Austria. The GRAL can model a wide range of spatial scales, from street-level through whole cities to a state-wide scale. The model takes as inputs topography, including buildings and infrastructure, sources with their emission profiles, and wind fields. The output is a spatiotemporal dense pollution map over the study region [32]. To compute this map and given the topography, the model takes into consideration building’s downwash effects through microscale modeling. The sources may be of different types, including surface road networks and point sources, such as tunnel ventilation outlets and industrial stacks, tunnel portals, and area sources. GRAL has been used extensively in regulatory assessments and scientific studies, such as calculating the impacts of road traffic or industry on air pollution, and it has been extensively validated in several different countries and contexts [30].

It is important to note that, with respect to meteorology, the GRAL model takes as input solely the wind field and does not regard any other chemical reactions and transformations between substances [33]. To this end, the wind field is computed by the GRAMM meteorological model, which is based on the Reynolds-averaged Navier–Stokes equations (RANS equations) and the law of mass conservation [34]. Thus, the term meteorology is limited to the wind field and its stability class. 

**Figure 1:**
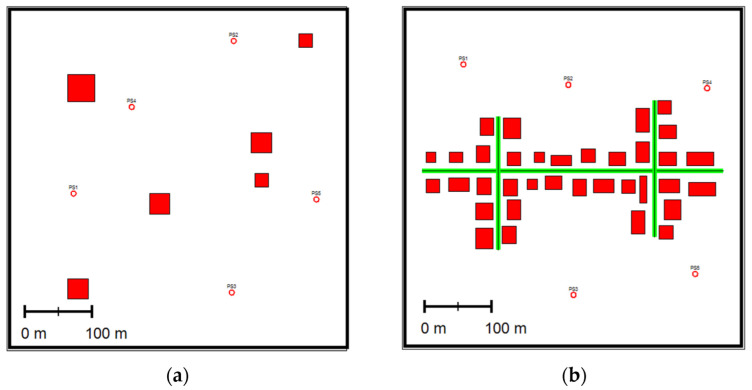
Schemas of the two scenarios: (**a**) small neighborhood, SNBH; (**b**) CBD, where the red squares indicate buildings; the green lines indicate line sources; and the circles indicate point sources (PS).

The region of interest, Ω, was modeled as a flat 500×500 m area divided into a grid of 5 × 5 m cells, {ω}∈Ω. In this area, two different scenarios were examined to present the capability of the method for a wide range of substances (gaseous and particulate matter) and topographical complexities:

1. A Small NeighBorHood, SNBH, consists of six buildings of different sizes and five-point sources emitting PM10 at different rates.
2. A Central Business District, CBD, consists of 35 buildings, three-line sources (i.e., roads), and five-point sources. ${\mathrm{NO}}_{x}$ was emitted from the point sources and the roads at different rates. Figure 1 depicts the two computer-generated scenarios.

For each meteorological condition (a given wind speed and direction), a dense pollution map was computed, where each grid point was represented by several pollution values, each of which corresponded to a specific meteorological condition. 

The meteorology input for the GRAL simulation was obtained from real-life data collected by the Israel Meteorology Service at the Hadera Port station (lon: lat, 34.8815: 32.4732) on 1 May 2020. For T, the total time in hours of the analysis, the temporal resolution of the meteorological data *in minutes* is ΔΦ; thus, for a T=24 h period, the number of samples was: $\left|q\right|=T\cdot \frac{60}{\Delta \Phi }$. Here, T=24, ΔΦ was a 10-min interval, so that |q|=144. The meteorological input for the GRAL simulation also contained the atmospheric stability classes (A–G), which were computed based on an atmospheric stability classification scheme [35].

The GRAL building prognostic approach was used with the default parameters. The maximum number of iterations for the internal flow field solver was 500 iterations, and concentration levels were measured three meters above ground level. A complete list of the parameter sets used for the GRAL computations is provided in the Supplementary Materials.

### 2.2. System Overview

When addressing the location–allocation problem in a given region for the first time, there is likely to be little information on pollution behavior in that specific region. On the other hand, information on the *static* attributes of the land, such as topography, land use, meteorological, and the locations of buildings, and potential pollution sources are more readily available. Thus, relying on static factors constitutes a more feasible approach. While we assume that potential sources’ locations are known a priori, the method suggested here is still applicable when sources’ locations are not known. This is discussed in the discussion section. Using *static* factors facilitated problem formulation, as described below.

In the initial stage, it is assumed that all sources emitted at a constant and equal rate (zero approximation). In this work, a constant rate of 100 kg/h was chosen arbitrarily. Then, a simulation generates a dense pollution map describing the pollution level, $C_{\omega }^{q}$, for meteorological condition q at location ω∈Ω. This process resulted in |*q*| different maps. Based on these |*q*| dense pollution maps, the locations that maximized a decision criterion were selected, resulting in a set of several possible deployment configurations. These possible configurations were evaluated for their ability to locate and quantify the source term under the simplistic zero approximation. Then, the optimal configuration was tested on other, more realistic source terms. 

### 2.3. Algorithmic Approach

Based on the |*q*| dense pollution maps generated by the simulation for the different meteorological conditions, under the zero-approximation assumption, for each location ω∈Ω, we obtained a set of measurements $\left\{C_{\omega }^{q}\right\}$. The measuring units of $\left\{C_{\omega }^{q}\right\}$ for gaseous matter can be parts per million (ppm), parts per billion (ppb) or $\mathrm{\mu gr}/m^{3}$; for particulate matter it can be particle number or $\mathrm{\mu gr}/m^{3}$. Regardless, it is important to note that the method and entropy measure are invariant to the measuring units. The analysis of these sets constituted the decision criterion, $DM_{\omega }\;\forall \omega \in \Omega$, to allocate the sensors. For $P\left(C_{\omega }^{q}\right)$ the empirical probability function of $C_{\omega }^{q}$, two different metrics were compared—Entropy (Equation (1)) and Hot Spot (Equation (2)):

(1) $$DM_{\omega }=-\underset{q}{\sum }P\left(C_{\omega }^{q}\right)\log P\left(C_{\omega }^{q}\right)$$

(2) $$DM_{\omega \in \Omega }=\underset{q}{\sum }C_{\omega }^{q}$$

The method is based on finding a set of sensor positions that maximize the entropy or hot spot score and have the lowest correlations between the sensor readings over time. The correlation between the two sensors is defined as the Pearson correlation index between the pollution concentration sets of the different meteorological conditions. This is accomplished using an iterative algorithm allocated to one sensor in each iteration. The following notations are used to describe the algorithm: let $d_{max}$ and d be the maximum available sensors for deployment and the number of sensors already deployed by the algorithm, respectively, and $\Omega^{d}\subseteq \Omega$ is the set of locations with sensors. For each location ω∈Ω, the set of neighboring locations is $B^{\omega }$. The union of all the cells’ neighboring sensors is then denoted by $B^{d}$. $B^{d}$ allows for avoiding placing sensors too close to each other, which would have represented the same information. Note that $B^{d}$ is automatically updated as $\Omega^{d}$ is updated.

Using the notation above, at each iteration, the candidate locations for placing a sensor are $\Omega^{N}=\Omega \backslash \left(\Omega^{d}\cup^{\;}B^{d}\right)$. For *N*, the maximum candidate locations for placing a sensor in each iteration, the locations are selected by taking the minimum between $\left|\Omega^{N}\right|$ and N locations in $\Omega^{N}$ with the highest DM score. Using small N values leads to sensor allocations mainly influenced by the *DM* score, whereas using large N values leads to sensor allocations mainly influenced by the correlation. A specific location is then selected from this set of candidate locations by computing the correlation between each candidate location and previously selected sensing locations and choosing the location with the lowest cumulative correlation. Here for each $d\leq d_{max\;}$, the *N* producing the best results is used. Typically, the *N* values were between 30 and 50. The algorithm appears in the box below (Algorithm 1):
**Algorithm 1:** Placing $d_{max}$ sensors with the highest *DM* score and the lowest correlationSet:               $\Omega^{d}=\phi$
Compute $DM_{\omega }\;\forall \omega \in \Omega$Place the first sensor in the location with the highest *DM* score,             $\omega^{*}=\underset{\omega \in \Omega }{\max}DM_{\omega }$Set:
$\Omega^{d}\leftarrow \omega^{*}$$d\leftarrow d_{max}-1$While d>0 do:
Find $\left\{\min\left(N,\left|\Omega \backslash \left(\Omega^{d}\cup^{\;}B^{d}\right)\right|\right)\right\}$ candidate locations in all optional locations, $\Omega \backslash \left(\Omega^{d}\cup^{\;}B^{d}\right)$, with the highest *DM* score:       $\Omega^{N}=\underset{\begin{array}{c} \omega \in \Omega \backslash \left(\Omega^{d}\cup^{\;}B^{d}\right)\\ \left|\Omega^{N}\right|=\min\left(N,\left|\;\Omega \backslash \left(\Omega^{d}\cup^{\;}B^{d}\right)\right|\right) \end{array}}{\max}DM_{\omega }$For each location in $\omega^{n}\in \Omega^{N}$, compute the correlation of $C_{\omega }^{q}$ with all locations where sensors have already been placed; i.e., $\omega^{d}\in \Omega^{d}$.Place the next sensor in location $\omega^{*}$ within $\Omega^{N}$, which presents the lowest summation of correlations with all locations in $\Omega^{d}$:        $\omega^{*}=\underset{\omega^{n}\in \Omega^{N}}{\min}\underset{\omega^{d}\in \Omega^{d}}{\sum }corr\left(C_{\omega^{d}}^{q},C_{\omega^{n}}^{q}\;\right)$Set:         i. $\Omega^{d}\leftarrow \Omega^{d}\cup^{\;}\omega^{*}$         ii. $d\leftarrow d_{max}-1$

Here $d_{max\;}$ was arbitrarily set to 30 and $B^{\omega^{d}}$, the restricted area in which sensors could not be placed around already deployed sensors, was a rectangular area of 4900 square meters (70 m×70 m), centered at $\omega^{d}$.

### 2.4. Deployment Evaluation

#### 2.4.1. Formulation

The deployment evaluation was conducted using the notation in Nebenzal et al. [36]. Recall that {D} is the set of sensors and {S}, a set of sources. d∈{D} is then a sensor located at $\omega^{d}\in \Omega$. For atmospheric conditions *q*, *d* records a pollution level of $C_{\omega^{d}}^{q}$. The source s∈S, located at $\omega^{s}\in \Omega$, emits at a rate of $r^{s}$. $m_{ds}^{q}$ is the pollution transfer function, which associates sensor *d*’s readings, located at $\omega^{d}\in \Omega$, with the emission of source *s* at atmospheric condition *q*:

(3) $$C_{\omega^{d}}^{q}=m_{ds}^{q}\cdot r^{s}$$

For a multiple source scenario, each sensor’s reading consists of the contribution of all sources, i.e.,:

(4) $$C_{\omega^{d}}^{q}=\underset{s\in \left\{S\right\}}{\sum }m_{ds}^{q}\cdot r^{s}$$

For the set {*D*}, the sources’ contributions for each of the sensors can be written in a matrix form:

(5) $$\vec{C^{q}}=M^{q}{\vec{r^{s}}}^{t}$$

where $\vec{C^{q}}$ is the row measurement vector, $M^{q}$ is the transfer matrix consisting of $m_{ds}^{q}$, and ${\vec{r^{s}}}^{t}$ is a column vector of the pollution emission rates. $M^{q}$ can be inferred either through empirical measurements or through a dispersion model.

Sensor deployment is then evaluated based on its ability to predict the source term; i.e., the real emission vector, $r^{s}$. This is achieved by formulating an optimization problem for a given meteorological condition, *q*:

(6) $$\begin{array}{cc} \underset{\vec{\hat{r}}}{\min} & ||M^{q}\vec{\hat{r}}-\vec{C^{q}}||\\ s.t & all\;elements\;in\;\hat{r}\geq 0 \end{array}$$

The solution to Equation (6) provides $\vec{\hat{r}}$, which represents the estimation of the source term, $r^{s}$, and thus its quality, is given by the normalized difference between the estimation of the source term and the true emission vector:

(7) $$E=\frac{||\vec{\hat{r}}-\vec{r^{s}}||}{||r^{s}||}$$

The computation of $\vec{\hat{r}}$ is repeated for every condition, *q*. In this case, 144 meteorological conditions are used, resulting in 144 source-term vector estimates. The element-wise median is taken as the emission rate of each source in the vector.

#### 2.4.2. Source Term Estimation

The zero approximation is a single value source term vector; i.e., $c\cdot \vec{1}$, with *c* arbitrarily set to 100 kg/h. Hence, sensor placement is based on a simplified source term vector. However, the evaluation was carried out using more realistic emission profiles that serve to evaluate the capability of the network to estimate complex source term vectors. Four configurations were used, one for the SNBH and three for the CBD scenarios, as listed in Table 1. Note that the 5th point source (PS5) in SNBH and CBD.1 is zero. Thus, not all potential sources need to be active.

#### 2.4.3. Comparison of Deployment Methods

Three deployment methods are evaluated in this study: random deployment and the application of Algorithm 1, using either the hotspot approach or the entropy to compute the DM score. The random deployment is justified because sensor installation is often governed by the availability of infrastructures, such as public facilities, power sources, utility poles, and communication towers. Random deployment is evaluated for each atmospheric condition, *q*, for $1\leq d_{max}\leq 30$. In these cases, for a given d the only two limitations on sensor placement are to avoid non-vacant grid points and to satisfy the “Box-Out” criterion; i.e., the possible candidate locations have to satisfy $\omega \in \Omega \backslash \left(\Omega^{d}\cup^{\;}B^{d}\right)$. Once the $d_{max}$ sensors are placed, deployment is evaluated using the method detailed above. Since this is a random process, the average error is computed for several different deployments (in this work, 50).

## 3. Results and Discussion

### 3.1. Dense Pollution Maps

**Figure 2:**
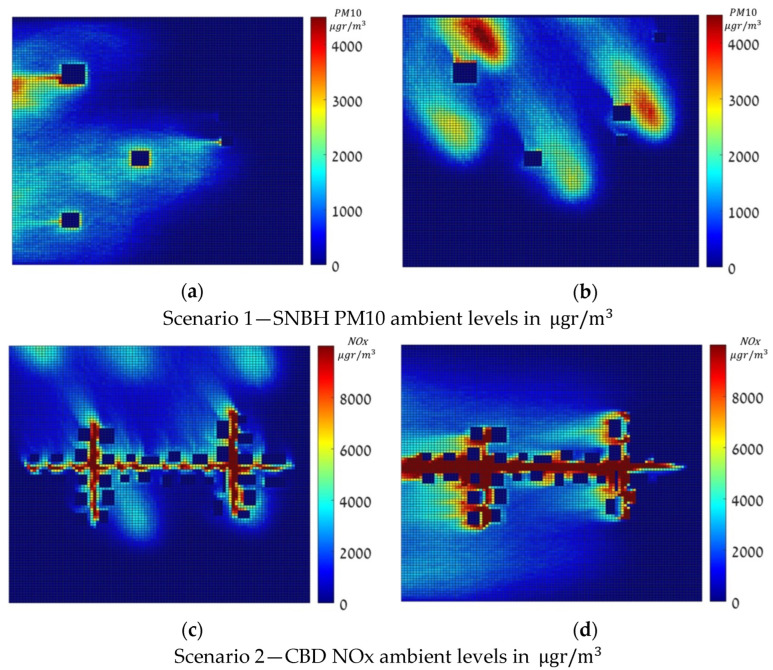
Dense Pollution Maps, in $\mathrm{\mu gr}/m^{3}$, under a zero-approximation emission vector.

The dense maps were computed for all 144 meteorological conditions extracted from a real dataset in Hadera, Israel, using the GRAL/GRAMM Lagrangian atmospheric dispersion model. Figure 2 shows four dense pollution maps in $\mathrm{\mu gr}/m^{3}$, for the two scenarios under a zero-approximation emission vector, where (a) and (b) depict the pollution over SNBH in two different meteorological conditions, and (c) and (d) over CBD in two other meteorological configurations. Note that the color scale is different for the SNBH and CBD scenarios. Note that these values, as defined Equation (2), are cumulative rather than instantaneous values.

### 3.2. Decision Matrix

**Figure 3:**
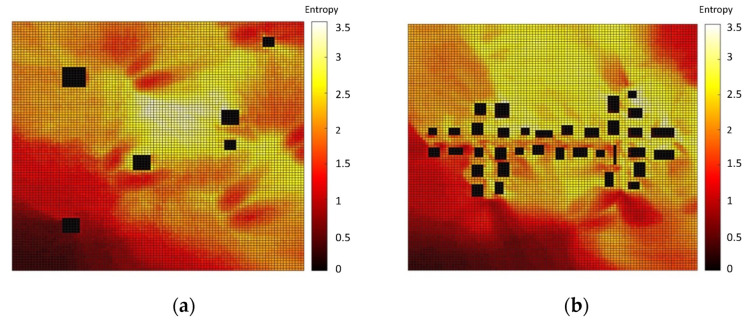
Entropy decision measures for the SNBH (**a**) and CBD (**b**) scenarios. Entropy has no units.

**Figure 4:**
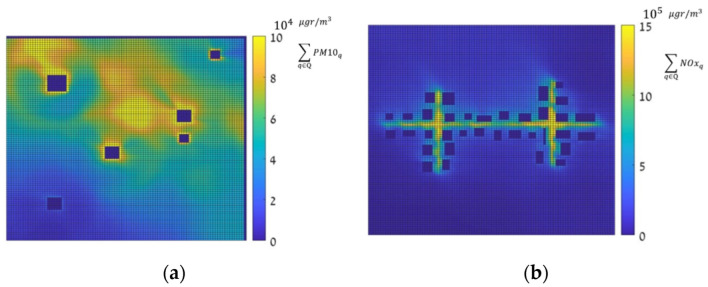
Hot spot decision measure, in $\mathrm{\mu gr}/m^{3}$, for the SNBH (**a**) and CBD (**b**) scenarios. Note that the color scale is different for figure (**a**,**b**).

Based on the 144 dense pollution maps, two types of decision matrices, Entropy (Equation (1)) and hotspot (Equation (2)), were computed. The entropy and hotspot decision matrices are presented in Figure 3 and Figure 4, respectively, where the right-hand side (a) in both figures represents the decision matrix for the SNBH, and the left-hand side shows the same matrix for CBD. It shows that for the SNBH scenario, similar patterns are obtained for both methods, whereas for the CBD scenario, each metric resulted in a different pattern. Hence, the data gain did not necessarily coalign with the locations with the highest pollution.

### 3.3. Sensor Placement

**Figure 5:**
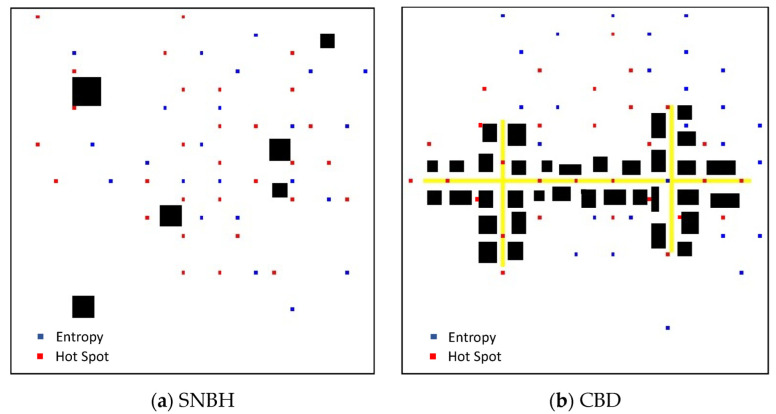
Optimal deployment as computed by Algorithm 1 for the SNBH (**a**) and CBD (**b**) configurations. The locations identified by the Entropy DM are marked in blue, while the locations identified by the hotspot DM are marked in red.

The optimal deployments of 30 sensors, as dictated by Algorithm 1 for the two DMs, entropy and hotspot, are presented Figure 5 The locations identified by the entropy DM are marked in blue, while the locations identified by the hotspot DM are marked in red. Comparing the two deployments, while considering Figure 3 and Figure 4 show that the main difference between the two deployments is that while the hotspot assigns the locations in the epicenter of the pollution field, the entropy allocates locations with larger pollution field gradients. This conclusion co-aligns with the conclusion reached by Kendler and Fishbain [37].

**Figure 6:**
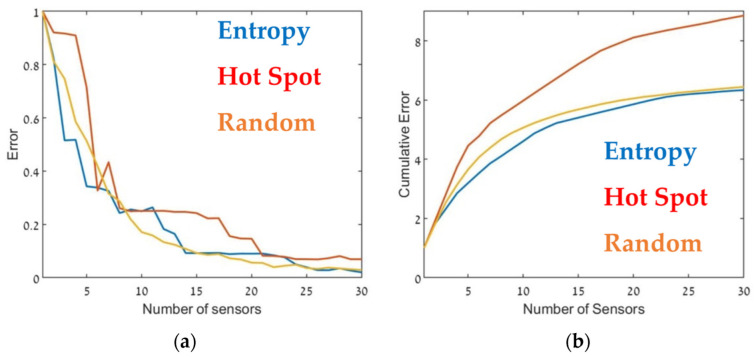
Error (Equation (7)) as a function of the number of sensors, $d_{max}$ (**a**) and the cumulative error (**b**) for SNBH. Entropy in blue, hot spot in red, and random in orange.

**Figure 7:**
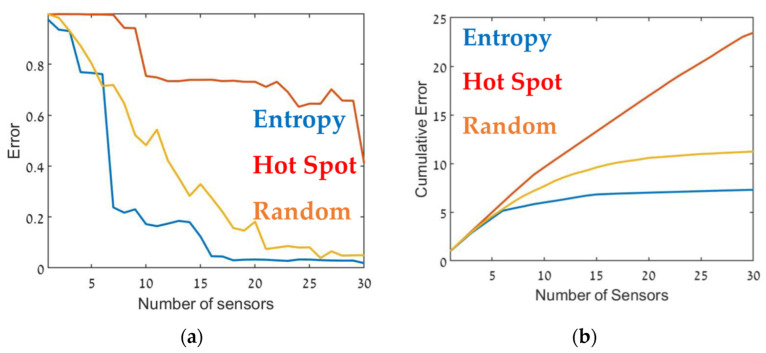
Error as a function of the number of sensors, $d_{max}$ (**a**) and the cumulative error (**b**) for the CBD.1 configuration (see Table 1). Entropy in blue, hot spot in red, and random in orange.

**Figure 8:**
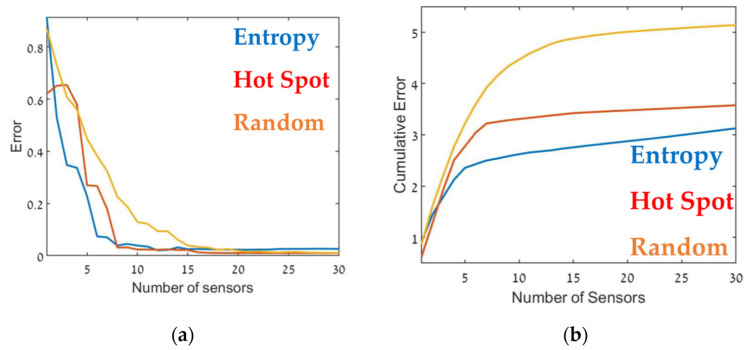
Error as a function of the number of sensors, $d_{max}$ (**a**) and the cumulative error (**b**) for the CBD.2 configuration (see Table 1). Entropy in blue, hot spot in red, and random in orange.

The optimal sensor deployment was assessed in terms of source-term estimation accuracy (Equation (7)). Figure 6, Figure 7 and Figure 8 show the error of the source term estimation for each of the deployment methods (entropy, hot spot, and random) as a function of $d_{max}$, for each SNBH, CBD.1, CBD.2, and CBD.3 scenario, as detailed in Table 1. The graphs show the error (left) and the cumulative error (right) as a function of the number of sensors. The graphs clearly indicate that, in all cases, the error did not reach stagnation and decreased steadily with each sensor added to the array in a similar way for all three metrics. The cumulative error for the entropy metric deployment was slightly better for each additional sensor deployed.

Although, in general, similar results were obtained in all cases, there were some interesting differences. In the case of CBD.1, where the point sources were dominant, with larger emission rates by one order of magnitude than the line sources, the Entropy metric significantly outperformed the hotspot metric. When the line sources were two orders of magnitude higher than the point sources (CBD.2), the difference between the entropy and hotspot metrics was less pronounced. In the case of the CBD.3 scenario in which the line source emission rate was only one order of magnitude higher than the point source, the entropy metric was marginally superior for sensor arrays comprised of fewer than 10–12 sensors. Increasing the number of sensors beyond 15 decreased the error when using the entropy metric but only led to a minor improvement in the hotspot metric. This difference was most noticeable for large sensor arrays, where the error for the entropy metric was five times lower than the hotspot metric. These findings suggest that the entropy metric was superior to the other two methods since it provided lower error and evidenced greater stability to changes in the site.

**Figure 9:**
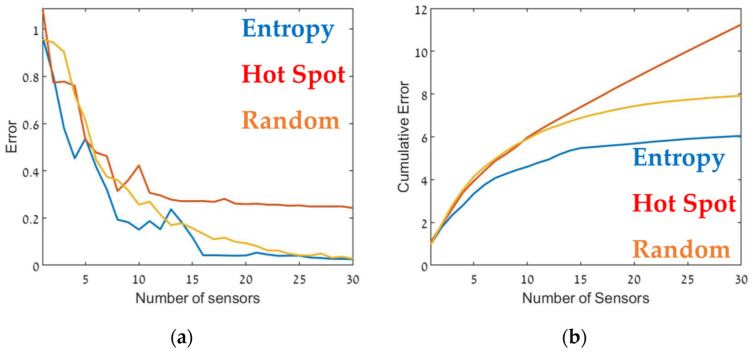
Error as a function of the number of sensors, $d_{max}$ (**a**) and the cumulative error (**b**) for the CBD.3 configuration (see Table 1). Entropy in blue, hot spot in red, and random in orange.

Table 2 presents the cumulative error obtained from each case for the maximum number of sensors, i.e., the overall error. The table suggests that in all cases, using the entropy metric for deploying the sensors resulted in an overall lower error compared to the hot spot or random deployment metrics. In certain specific cases, for example, Figure 9, for 12–13 sensors, the random or hot spot metric emerged as slightly better than entropy, but the overall trend was clear. Further, using the hot spot metric resulted in inconsistent performance, where in some cases the results for this metric were similar to those obtained using entropy (for example, Figure 8), but in others was considerably worse (Figure 7).

Thus, Figure 6 through Figure 9 and Table 2 show that the entropy approach produced superior accuracy and stability over the hot spot or random methods. These findings suggest that sensors should be placed in locations where the information gain is maximal. In the entropy method, informativity is based on a concept taken from information theory, where the entropy of a random variable is the average level of information inherent in the variable’s possible outcomes. In this case, the random variable was the pollution level at each grid point, and its possible outcomes were all possible readings of the air pollution sensor.

## 4. Conclusions

This study presents a new information theory-based approach to the deployment of air pollution sensors. This entropy-based approach was compared to methods based on hot spots and random metrics. The random approach simulated situations in which no previous knowledge was available, and the deployment was mainly dictated by availability and convenience.

The presented method does not require any prior knowledge of the number and source locations, since the optimization problem (Equation (6)) can be solved for sources with a pollution emission rate that equals zero. The ability of the method here to estimate a source with zero emissions (see source PS5 in CBD.1 in Table 1) makes it possible to set the source term vector to be the size of the entire region of interest; thus, $\left|\vec{\hat{r}}\right|=\left|\Omega \right|$ and a source can be in each ω∈Ω. In this fashion, locations without a source are estimated to be zero. A similar notion was presented by Nebenzal et al. [36,38]. The study here addresses optimal sensor deployment in a small neighborhood. The algorithm can be easily adapted to a large-scale deployment in a city and even on a national scale.

Future work will include the extension of this work to cases where the number and locations of the sources are unknown, where the simulations will be run with real sensor capabilities (sensitivity and dynamic range), and a comparison to field experiments will be made. The application of the methodology over a large geographical scale will also be sought.

## Figures and Tables

**Table 1:** Emission rates from different sources in different scenarios.

			PS1	PS2	PS3	PS4	PS5	Line 1	Line 2	Line 3
SNBH		Emission rate: [kg/h]	100	50	200	100	0	N/A	N/A	N/A
CBD	CBD.1		150	100	50	200	0	10	15	20
	CBD.2		5	3	9	7	10	1000	1000	1000
	CBD.3		50	30	90	70	10	100	200	150

**Table 2:** The cumulative error in each scenario for each deployment metric. Best result, for each scenario (SNBH, CBD.1, CBD.2 and CBD.3), are highlighted in bold.

	Entropy	Hot Spot	Random	Max Random
SNBH	**6.34**	8.85	6.44	13.64
CBD.1	**7.29**	23.45	11.23	25.14
CBD.2	**3.13**	3.58	5.14	12.77
CBD.3	**6.04**	11.25	7.92	23.35

## Data Availability

Not applicable.

## Supplementary Materials

The following supporting information can be downloaded at: https://www.mdpi.com/article/10.3390/s22103808/s1, Table S1: List of parameters used for GRAL computation.
