# Supplementary material for: Information Theory Solution Approach to the Air Pollution Sensor Location–Allocation Problem
Source: Sensors (Basel). 2022 May 17;22(10):3808. doi: 10.3390/s22103808 (PMC9147153; doi:10.3390/s22103808)
Supplement: Supplementary file 1 [file sensors-22-03808-s001.zip › sensors-1668450-supplementary.pdf]

sensors

MDPI

Supplementary Information

Table S1: List of parameters used for the GRAL computation.

	Parameter	Value	Units
Operational	Operation duration	86,400	Second
Parameters	Dispersion time	3600	Second
	Particles per second	100	Second/1
Meteorology	Surface roughness	0.2	m
	Roughness of building walls	0.01	m
	Horizontal grid resolution	300	m
	Vertical thickness of first layer	2	m
	Vertical stretching factor	1.01	
	Number of cells in z direction	40	
	Min/max number of iterations	100-1000	
Scene #1			
PS1	Source diameter	0.6	m
	Exit temperature	298	K
	Emitted gas	$PM10$	
	Deposition		
	Exit velocity	2	m/s
	Height	25	m
PS2	Source diameter	0.6	m
	Exit temperature	298	K
	Emitted gas	$PM10$	
	Deposition		
	Exit velocity	1.5	m/s
	Height	25	m
PS3	Source diameter	0.6	m
	Exit temperature	323	K
	Emitted gas	$PM10$	
	Deposition		
	Exit velocity	2.5	m/s
	Height	25	m
PS4	Source diameter	0.5	m
	Exit temperature	303	K
	Emitted gas	$PM10$	
	Deposition		
	Exit velocity	3	m/s
	Height	20	m
PS5	Source diameter	0.6	m
	Exit temperature	298	K
	Emitted gas	$PM10$	
	Deposition		
	Exit velocity	2	m/s
	Height	20	m
Scene #2			
PS1	Source diameter	0.6	m
	Exit temperature	323	K

	Emitted gas	NO_x	
	Deposition		
	Exit velocity	2	m/s
	Height	20	m
PS2	Source diameter	0.5	m
	Exit temperature	323	K
	Emitted gas	NO_x	
	Deposition		
	Exit velocity	3	m/s
	Height	25	m
PS3	Source diameter	0.6	m
	Exit temperature	393	K
	Emitted gas	NO_x	
	Deposition		
	Exit velocity	2.5	m/s
	Height	20	m
PS4	Source diameter	0.6	m
	Exit temperature	303	K
	Emitted gas	NO_x	
	Deposition		
	Exit velocity	2	m/s
	Height	20	m
PS5	Source diameter	0.6	m
	Exit temperature	303	K
	Emitted gas	NO_x	
	Deposition		
	Exit velocity	2	m/s
	Height	20	m
Line1/Line2/Line3	Width	7	m
	Emitted gas	NO_x	
	Vertical exit	3	m
	Height	0	m
